# Association of Anti-TPO Antibody and Inflammatory Markers with Thyroid Ultrasound Findings

**DOI:** 10.2174/0118715303429924250912052941

**Published:** 2025-09-18

**Authors:** Ersin Kuloglu, Kubilay Issever, Ali Muhtaroglu, Sefer Aslan, Berkan Acar

**Affiliations:** 1 Department of Internal Medicine, Faculty of Medicine, Giresun University, Giresun, Turkey;; 2 Department of General Surgery, Faculty of Medicine, Giresun University, Giresun, Turkey

**Keywords:** Subclinical hypothyroidism, anti-thyroid peroxidase antibody (anti-TPO), thyroid sonography, inflammation markers, nodule characteristics, demographic data

## Abstract

**Introduction:**

The objective of this study was to evaluate the demographic, clinical, laboratory, and ultrasonographic characteristics of patients diagnosed with subclinical hypothyroidism, with a particular emphasis on the anti-thyroid peroxidase (anti-TPO) antibody and inflammatory biomarkers.

**Methods:**

The study included 157 patients diagnosed with subclinical hypothyroidism, categorised into anti-TPO-positive and anti-TPO-negative groups. A retrospective comprehensive evaluation comprising demographic data, thyroid medication status, ultrasonographic characteristics, and laboratory parameters was conducted and statistically analysed between the groups.

**Results:**

Of 157 patients, 48.4% were anti-TPO positive. This group was significantly associated with increased levothyroxine (LT4) use and sonographic parenchymal heterogeneity. However, there were no significant differences in nodule presence, number, size, or structure. A positive correlation was found between anti-TPO and ferritin levels. In addition, a positive correlation was observed between the thyroid-stimulating hormone (TSH)/free T4 ratio and the solidity of nodules, as well as between TSH and the neutrophil-to-lymphocyte ratio (NLR). Surprisingly, a negative correlation was found between anti-TPO levels and the number of nodules, as well as the cystic characterisation of the nodules.

**Discussion:**

In our study, higher levels of anti-TPO and TSH were associated with inflammatory markers such as ferritin and NLR, suggesting a possible link with systemic inflammation. Furthermore, anti-TPO and the TSH/T4 ratio also showed associations with specific sonographic features of the thyroid gland.

**Conclusion:**

TSH and anti-TPO levels might be associated with systemic inflammation and thyroid sonographic findings in patients with subclinical hypothyroidism. More studies on larger patient populations should confirm the same results to suggest their clinical significance.

## 1 INTRODUCTION

The thyroid gland, a vital endocrine organ situated anteriorly in the neck, has a significant influence on various metabolic processes by primarily secreting thyroxine (T4) and, to a lesser extent, triiodothyronine (T3). Although the thyroid gland predominantly produces T4, peripheral tissues convert T4 into the more biologically active T3, highlighting the systemic role of these hormones beyond thyroid secretion alone [1, 2]. In addition to regulating metabolic rate, these hormones modulate several other physiological processes, including cardiac function, thermogenesis, gastrointestinal activity, and overall growth and development [3, 4]. Dysfunctions in thyroid hormone production result in clinical conditions ranging from hypothyroidism, characterised by inadequate thyroid hormone levels, to hyperthyroidism, characterised by excessive hormone production. Subclinical hypothyroidism is a prevalent thyroid disorder, characterised by elevated serum thyroid-stimulating hormone (TSH) levels in the presence of normal circulating thyroid hormone levels. A wide range of underlying causes has been identified, including autoimmune thyroiditis, particularly Hashimoto's thyroiditis, iodine deficiency or excess, certain medications, and previous treatments affecting the thyroid gland, such as radioactive iodine therapy or surgery [5].

A significant diagnostic and prognostic biomarker in autoimmune thyroid disorders is the anti-thyroid peroxidase (anti-TPO) antibody. It has been established that anti-TPO antibodies can target the thyroid peroxidase enzyme, which is crucial for the oxidation of iodine and the subsequent synthesis of thyroid hormones. The presence of these antibodies has been demonstrated to be strongly associated with autoimmune thyroid disease, reflecting underlying chronic inflammation and glandular injury [6].

Inflammation markers, such as the neutrophil-to-lymphocyte ratio (NLR), systemic immune-inflammatory index (SII: platelet × neutrophil/lymphocyte), pan-immune inflammation value (PIV: platelet × neutrophil × monocyte/lymphocyte), and ferritin, have gained prominence as significant indicators of systemic inflammation. Elevated levels of these markers may reflect ongoing inflammatory processes associated with autoimmune thyroid disease and thyroid dysfunction, suggesting a potential predictive role for these conditions [7].

This study investigates the demographic, clinical, biochemical, and ultrasonographic characteristics associated with anti-TPO antibody positivity in patients with subclinical hypothyroidism. Another objective of the study is to identify serum biomarkers that have the capacity to predict sonographic findings for risk stratification in health care facilities where ultrasonography is not available.

## 2 MATERIALS AND METHODS

### 2.1 Ethical Approval

The local ethics committee of Giresun Training and Research Hospital approved the study protocol with the decision numbered 13.11.2024/07. Due to the retrospective nature of the study, informed consent was waived. This study adhered to the ethical principles outlined in the 2013 revision of the Declaration of Helsinki.

### 2.2 Data Collection

This retrospective study included patients diagnosed with subclinical hypothyroidism between January 2024 and December 2024, identified through medical records and laboratory tests conducted at our internal medicine outpatient clinics. The inclusion criteria consisted of being 18 years or older, having elevated serum thyroid-stimulating hormone (TSH) levels with concurrently normal serum free T4 and T3 concentrations, and having comprehensive demographic, clinical, biochemical, and sonographic data available. Patients with a history of past or active thyroid malignancy, a history of thyroidectomy, previous radioactive iodine therapy, radiotherapy to the head and neck region, active malignancy, acute infection, chronic inflammatory disease, chronic liver, renal, or heart failure, and those under the age of 18 were excluded from the study. Demographic data, including age and gender, as well as information regarding thyroid medication use—specifically levothyroxine (LT4) treatment—were documented. LT4 treatment status was categorized based on medication dosage, duration, and adherence, as determined by patient medical histories and pharmacy records. In our study, levothyroxine usage data were unavailable for 4 patients.

Laboratory analyses involved evaluating serum concentrations of TSH (mIU/L), free T4 (ng/dL), anti-TPO antibodies (IU/mL), and ferritin (µg/L). Additionally, calculated inflammatory and thyroidal indices, such as NLR, SII, PIV, and the TSH/T4 ratio (mIU/L / ng/dL), were assessed to determine the systemic inflammatory status. Confounding factors that may influence the inflammatory status of patients with subclinical hypothyroidism, such as body mass index (BMI), lipid levels, iron status, nutritional characteristics, and history of smoking and alcohol consumption, could not be evaluated. Patients were classified into two groups based on their anti-TPO antibody status in the subgroup analyses: those with anti-TPO antibodies and those without.

Thyroid sonography was conducted using high-resolution ultrasound equipment operated by the same experienced radiologist. Sonographic parameters assessed included nodule presence, number, size classification (<1 cm or ≥1 cm), structure (solid or cystic), and overall parenchymal structure classified as homogeneous or heterogeneous according to guidelines established by the ATA and the TI-RADS. Thyroid nodules measuring ≥2 mm in minimum diameter were included in the study. Patients with different numbers and sizes of thyroid nodules were presented in separate groups to avoid overlapping.

### 2.3 Statistical Analysis

Comparative statistical analyses were conducted between these groups using appropriate methods. Categorical data comparisons utilised Chi-square tests, while continuous variables were compared using Independent T-Tests, depending on the data distribution. Correlation analyses were performed using Pearson’s correlation coefficients to explore relationships between inflammatory markers, thyroid function parameters, and sonographic findings. The effects of TSH/T4 ratios on the presence of solid nodules and the effects of anti-TPO levels on the presence of cystic nodules were evaluated using logistic regression analysis. In addition, the effects of anti-TPO levels on the number of nodules and ferritin levels, as well as the effects of TSH levels on NLR ratios, were analyzed using simple linear regression analysis. Statistical significance was defined as *p*-values less than 0.05. All statistical analyses were conducted using SPSS software (version 26.0, IBM Corporation, Armonk, NY, USA).

## 3 RESULTS

### 3.1 Demographic and Clinical Characteristics

A total of 157 patients diagnosed with subclinical hypothyroidism were included in this study. The mean age of the patients was 42.46 ± 16.53, and females accounted for 70.1% of the cohort. Information regarding the patients' gender, age, LT4 use, anti-TPO status, presence of thyroid nodules, number of nodules, size, and sonographic characteristics (solid or cystic), as well as thyroid parenchymal structure, is presented in Table **1**.

### 3.2 Comparison of Demographic and Clinical Characteristics of the Groups

Patients diagnosed with subclinical hypothyroidism were divided into two groups based on anti-TPO positivity or negativity. The groups were compared according to gender, age, LT4 use, presence of thyroid nodules, number of nodules, size, and sonographic characteristics (solid or cystic), as well as thyroid parenchymal structure (Table **2**). Patients with anti-TPO positivity significantly exhibited higher LT4 usage compared to those who were anti-TPO negative (*p* = 0.000). Ultrasonographic examination revealed that parenchymal heterogeneity was significantly more prevalent in patients with anti-TPO antibodies compared to those without (93.9% *versus* 58.2%, respectively; *p* < 0.05). However, no significant differences were observed between the anti-TPO-positive and negative groups regarding the presence of nodules, the number of nodules, their size, or characteristics (*p* > 0.05).

When these two groups were compared in terms of laboratory parameters and novel inflammatory indices, no significant differences were observed in the parameters such as TSH, PIV, NLR, SII, and ferritin.

### 3.3 Correlation Analysis of the Parameters

The results of the correlation analysis between the laboratory and sonographic findings of patients with subclinical hypothyroidism are presented in Table **3**. In our study, a negative correlation was observed between anti-TPO levels and nodule number and the presence of cystic nodules. A positive correlation was identified between the TSH/T4 ratio and the presence of solid nodules. Additionally, positive correlations were found between TSH levels and NLR, as well as between anti-TPO and ferritin levels. Furthermore, correlation analyses among other parameters are presented in Table **3**.

### 3.4 Regression Analysis of the Correlated Parameters

The findings of the regression analyses are presented in Table **4a** and **4b** . Table **4a** demonstrates the logistic regression analysis results of factors associated with the presence of solid and cystic nodules, while Table **4b** presents the linear regression analysis results showing the associations of anti-TPO and TSH with nodule number, ferritin, and NLR.

## 4 DISCUSSION

In this study, the association between anti-TPO antibody positivity and various demographic, clinical, biochemical, and ultrasonographic parameters was investigated in patients with subclinical hypothyroidism. The findings of the present study demonstrated that anti-TPO levels were correlated with fewer nodules and a lower frequency of cystic morphology, and the TSH/T4 ratio was correlated with a higher frequency of solidity in the sonographic findings. Regarding inflammation, anti-TPO levels were correlated with ferritin levels, while TSH levels were correlated with NLR. These important results might open new chapters in the book of subclinical and autoimmune hypothyroidism, especially given the fact that the literature contains less research regarding these associations.

The analysis revealed a significant correlation between the positivity of anti-TPO antibodies and higher rates of LT4 prescription. This finding is consistent with the conclusions of previous studies, which have indicated that the presence of anti-TPO positivity is a reliable predictor of the development of overt hypothyroidism, suggesting the need for therapeutic intervention [8, 9]. The identification of patients requiring LT4 replacement at earlier stages through anti-TPO antibody screening may enable clinicians to adopt a more proactive management approach.

Expectedly, sonographic parenchymal heterogeneity was found to be significantly more prevalent in patients with anti-TPO positivity compared to those with anti-TPO negativity. This observation is consistent with the pathophysiology of autoimmune thyroiditis, where chronic inflammation leads to diffuse glandular damage, reflected as heterogeneous parenchyma on ultrasonography [10, 11]. However, the study's results revealed no significant differences between the groups in terms of nodule prevalence, size, number, or structure (solid or cystic). This finding is corroborated by existing research, which suggests that thyroid nodularity is not necessarily influenced by autoimmune mechanisms, but rather by factors such as iodine intake and genetic predisposition [12, 13]. Controversially, when anti-TPO levels were analyzed quantitatively, rather than positivity, a significant negative correlation was observed with nodularity and cystic morphology of the nodules. In patients who are anti-TPO positive, increased thyroid parenchymal heterogeneity may hinder the detection of small-sized thyroid nodules by sonographic methods. This represents a technical limitation of the sonographic method used in our study and can be considered as one of the reasons for these controversial results observed in our study. Considering the general opinion in the literature that autoimmune hypothyroidism is linked with increased nodularity and thyroid cancers, the potential reasons for this result should be discussed further and verified by more research with larger cohorts [14, 15].

‘Thyroglobulin is a large glycoprotein synthesized by thyroid follicular cells and secreted into the follicular lumen. In patients with thyroid cancer, an elevated level of thyroglobulin, produced by malignant cells, is observed. Several studies have demonstrated that measuring serum thyroglobulin levels plays a significant role in the initial screening of patients with thyroid nodules and in predicting cancer risk. Although we could not obtain the thyroglobulin levels of these patients from their medical records, it would be useful to analyze the association between thyroglobulin levels and sonographic characterization of the nodules to investigate this topic in future studies more comprehensively [16, 17].

It is noteworthy that no significant differences were observed in laboratory parameters or the level of inflammation between the anti-TPO-positive and anti-TPO-negative groups. When anti-TPO was analyzed quantitatively, the logistic regression analysis results demonstrated a significant correlation between anti-TPO antibody and ferritin levels. Upon reviewing the literature, it was observed that there were insufficient studies investigating the association of serum ferritin levels with anti-TPO antibody levels. In a study evaluating thyroid function tests, ferritin, and anti-TPO antibody levels in 143 patients, similar to our findings, a significant association was demonstrated between elevated anti-TPO antibody levels and increased ferritin concentrations. The significant association between anti-TPO antibody and ferritin levels is explained by the underlying immuno-inflammatory pathway [18]. A study was conducted in Iraq, comparing the thyroid function tests and serum ferritin levels of 50 patients with newly diagnosed Hashimoto's thyroiditis with those of a group of 40 healthy individuals. Unlike in our study, it was found that serum ferritin levels were lower in the Hashimoto thyroiditis group [19]. Patients with subclinical hypothyroidism should be evaluated in terms of possible accompanying iron deficiency and nutritional status in addition to autoimmune-inflammatory markers [18]. In another retrospective study comparing patients according to their ferritin levels (low, normal, and high ferritin), no significant differences were found in anti-TPO levels between the groups [20]. At this point, it is important to remind that the anti-TPO antibody has certain limitations in the follow-up of patients with hypothyroidism. In patients with long-standing hypothyroidism, anti-TPO antibody levels may decrease over time due to thyroid gland destruction secondary to the chronic disease. This process reduces the utility of anti-TPO antibody as a marker for monitoring thyroid function and autoimmunity [21]. Considering these controversial results regarding the association between ferritin and anti-TPO levels, especially when the potential pathophysiological relationship between iron deficiency anemia and hypothyroidism is taken into account, the literature requires further studies to clarify this issue. If there is a consensus regarding the correlation between anti-TPO and ferritin levels according to future similar studies, this might affect the clinicians' daily practices by making them more cautious for patients with elevated anti-TPO levels. These patients can be followed up more closely for overt hypothyroidism and other conditions related to chronic inflammation of the body.

One of the striking results of our study is the positive correlation between the NLR and TSH levels. NLR has gained recognition as a surrogate marker for systemic metabolic inflammation, with elevated levels reported in various autoimmune and inflammatory diseases [22-24]. The elevated NLR and increased TSH levels observed in this cohort suggest a potential link between systemic inflammation and subclinical hypothyroidism, particularly in the context of autoimmunity. Furthermore, as the level of hypothyroidism increases, patients are more likely to have inflammation, according to this result. This correlation has also been demonstrated by recent investigations, which have shown that patients with chronic autoimmune thyroiditis often exhibit increased NLR values that correlate with disease activity and progression [25, 26]. Subclinical hypothyroidism patients with higher NLR values may be more prone to systemic chronic low-level inflammation and its potential complications, such as atherosclerosis and endothelial injury, similar to those observed in obesity [27]. This hypothesis must also be supported by more studies with larger patient populations.

Last but not least, the positive correlation between the TSH/T4 ratio and thyroid nodular solidity was also revealed in our study. As the TSH/T4 ratio increases, patients with subclinical hypothyroidism become closer to overt hypothyroidism. This result indicates that as the patients become closer to overt hypothyroidism, they are more likely to have solid nodules in their thyroid tissue. Multicenter studies with large patient populations are needed to clearly elucidate the relationship between anti-TPO and TSH/T4 levels and the sonographic findings of the thyroid gland.

While these inflammatory markers are not specific, they may serve as valuable tools in monitoring subclinical hypothyroidism, particularly when determining the likelihood of autoimmune involvement, disease progression, and LT4 replacement. The incorporation of these parameters into clinical algorithms has the potential to improve the stratification of patients for surveillance or early intervention. Further prospective studies with larger cohorts are required to clarify the relationships between systemic inflammatory responses, thyroid dysfunctions, and sonographic findings.

## CONCLUSION

Correlations between inflammatory markers and thyroid function may highlight the role of systemic inflammation in thyroid autoimmunity and dysfunction. Correlations between anti-TPO and TSH/T4 ratio and thyroidal sonographic findings might offer new clinical management strategies. The associations identified in our study can be considered as hypothesis-generating and may serve as a basis for future multi-center, longitudinal studies with larger patient cohorts. If the same results are observed in similar studies, it might be possible to predict the sonographic findings of these patients based on laboratory tests in settings such as primary care centers where ultrasound is not available.

## STUDY LIMITATION

Although the study demonstrates strengths, limitations in the research design and inferences must be acknowledged, as well as the potential for broader generalisations to be made. The retrospective and cross-sectional nature of the research, coupled with the absence of longitudinal data, limits the ability to draw definitive causal conclusions. The single-centre nature of the study and low R^2^ values in the logistic regression analysis limit its generalisability, and the findings may not fully reflect broader demographics or geographical differences. The utilisation of existing data sets necessitates the exclusion of inflammatory markers (*e.g*., CRP, IL-6, TNF-α), which could offer mechanistic explanations. While ferritin was used as an inflammatory marker, other factors (*e.g.*, iron status, viral load, nutritional status) may also have influenced the results, and these were not fully controlled. The parameters that could affect the inflammatory laboratory results of patients, such as smoking, alcohol consumption, body mass index, lipid levels, and medications used, were not available in the patient files. The inclusion of 157 patients, the retrospective and cross-sectional nature of our study, and the inability to fully account for confounding inflammatory factors limit the ability to draw definitive conclusions regarding the relationships between laboratory and ultrasonographic findings. The cross-sectional nature of the data prevents assessment of disease progression, response to treatment, or the significance of the inflammatory and imaging findings. Therefore, our results can be classified as hypothesis-generating rather than definitive clinical findings.

## Figures and Tables

**Table 1:** Demographic and clinical characteristics of patients with subclinical hypothyroidism.

Variables		Number	%
Gender	Female	110	70.1
-	Male	47	29.9
Use of levothyroxine (LT4)	Absent	111	72.5
-	LT4 25 mcg/day	9	5.9
-	LT4 50 mcg/day	13	8.5
-	LT4 75 mcg/day	10	6.5
-	LT4 100 mcg/day	10	6.5
Anti-TPO	Negative	81	51.6
-	Positive	76	48.4
Nodule presence	Absent	94	59.9
-	Present	63	40.1
Nodule number	Absent	94	59.9
-	1	29	18.5
-	2	9	5.7
-	3	6	3.8
-	Multiple	19	12.1
Number of nodules (size: <1 cm)	Absent	96	61.1
-	1	31	19.7
-	2	10	6.4
-	3	1	0.6
-	Multiple	19	12.1
Number of nodules (size: ≥1 cm)	Absent	137	87.3
-	1	15	9.6
-	2	5	3.2
Nodule structure: solid	Absent	148	94.3
-	Present	9	5.7
Nodule structure: cystic	Absent	121	77.1
-	Present	36	22.9
Parenchymal structure	Homogeneous	32	24.1
-	Heterogeneous	101	75.9
Age	Mean ± S.D. (Min.-Max.)	42.46 ± 16.53 (19-86)	

**Abbreviatons:** S.D.: Standard Deviation, Min.: Minimum, Max.: Maximum, LT4: Levothyroxine, anti-TPO: anti-thyroid peroxidase, Nodules with a diameter greater than 2 mm were included in the study. The largest diameter of each nodule was recorded. Patients with different numbers and sizes of thyroid nodules were presented in separate groups to avoid overlapping.

**Table 2:** Comparison of demographic and clinical characteristics of patients with subclinical hypothyroidism according to anti-TPO antibody positivity.

Variables		Anti-TPO Negative (n:81)		Anti-TPO Positive (n:76)		*p*
		Number	*%*	Number	*%*	
Gender	Female	58	71.6	52	68.4	0.794
-	Male	23	28.4	24	31.6	
Use of levothyroxine (LT4)	Absent	66	82.5	45	61.6	**0.000**
-	LT4 25 mcg/day	6	7.5	3	4.1	
-	LT4 50 mcg/day	4	5.0	9	12.3	
-	LT4 75 mcg/day	0	0.0	10	13.7	
-	LT4 100 mcg/day	4	5.0	6	8.2	
Nodule presence	Absent	47	58.0	47	61.8	0.745
-	Present	34	42.0	29	38.2	
Nodule number	Absent	47	58.0	47	61.8	0.633
-	1	14	17.3	15	19.7	
-	2	4	4.9	5	6.6	
-	3	3	3.7	3	3.9	
-	Multiple	13	16.0	6	7.9	
Nodule structure: solid	Absent	77	95.1	71	93.4	0.922
-	Present	4	4.9	5	6.6	
Nodule structure: cystic	Absent	59	72.8	62	81.6	0.266
-	Present	22	27.2	14	18.4	
Parenchymal structure	Homogeneous	28	41.8	4	6.1	**0.000**
-	Heterogeneous	39	58.2	62	93.9	-
-	-	Mean ± S.D. (Min.-Max.)		Mean ± S.D. (Min.-Max.)		***p***
Age^t^		42.84 ± 18.22 (19-86)		42.07 ± 14.64 (19-78)		0.769
Nodule number^t^		0.86 ± 1.19 (0-3)		0.68 ± 1.04 (0-3)		0.315

**Note:** *P* values that are statistically significant have been highlighted in bold for emphasis. **Abbreviations:** *x*^2^ Chi-square test (Categorical data), t: Independent Sample T-Test, LT4: Levothyroxine.

**Table 3:** Correlation analysis results between the laboratory and sonographic findings of patients with subclinical hypothyroidism.

Variables	PIV	SII	NLR	Ferritin	Anti-TPO (Quantitative Value)	TSH/T4 Ratio	TSH	T4
Age	-0.094	-0.037	0.072	**0.245**	-0.032	**0.171**	0.141	-0.136
Gender	0.145	0.063	0.078	**0.532**	0.058	0.082	0.121	0.157
TSH (mIU/L)	-0.021	0.063	**0.161**	-0.017	0.115	**0.954**	1.000	-0.024
T4 (ng/dL)	0.011	0.024	0.060	0.046	0.029	**-0.302**	-0.024	1.000
anti-TPO (IU/mL)	-0.049	-0.059	-0.028	**0.178**	1.000	0.110	0.115	0.029
PIV	1.000	**0.863**	**0.632**	**0.189**	-0.049	-0.015	-0.021	0.011
SII	**0.863**	1.000	**0.822**	0.161	-0.059	0.058	0.063	0.024
NLR	**0.632**	**0.822**	1.000	**0.223**	-0.028	0.141	**0.161**	0.060
TSH/T4 Ratio (mIU/L / ng/dL)	-0.015	0.058	0.141	-0.020	0.110	1.000	**0.954**	**0.302**
Ferritin (µg/L)	**0.189**	0.161	**0.223**	1.000	**0.178**	-0.020	-0.017	0.046
Nodule presence	-0.072	-0.071	-0.006	0.067	-0.135	0.034	0.006	-0.102
Nodule number	-0.056	-0.054	0.002	0.029	**-0.162**	0.033	-0.007	-0.126
Solid nodule	-0.014	-0.022	-0.023	0.028	-0.090	**0.166**	0.135	-0.112
Cystic nodule	-0.007	0.016	0.041	-0.113	**-0.189**	-0.004	-0.027	-0.047
Parenchymal structure	0.004	0.042	0.123	0.075	**0.278**	0.012	0.040	0.133

**Note:** *P* values that are statistically significant have been highlighted in bold for emphasis. **Abbreviations:** r: Correlation coefficient, TSH: Thyroid Stimulating Hormone, T4: Thyroxine, anti-TPO: anti-Thyroid Peroxidase Antibody, PIV: Pan-Immune-Inflammation Value, SII: Systemic Immune-Inflammation Index, NLR: Neutrophil-to-Lymphocyte Ratio.

**Table 4a:** Logistic regression analysis results of factors associated with the presence of solid and cystic nodules.

Dependent Variable	Independent Variable	B	S.E.	*p*	Exp(B)/ Odds Ratio	Confidence Interval 95 C.I.for EXP(B)		Nagelkerke R^2^
						Lower	Upper	
Solid nodule	TSH/T4 Ratio (per 10 units)	1.950	1.01	**0.050**	7.029	0.971	50.887	0.059
Cystic nodule	Anti-TPO (per 10 IU/mL)	-0.04	0.02	**0.028**	0.961	0.924	0.999	0.065

**Note:** *P* values that are statistically significant have been highlighted in bold for emphasis. **Abbreviations:** Exp(B), Odds Ratio (OR): Logistic regression analysis, B: Unstandardized path coefficient, β (Beta): Standardized path coefficient, R^2^(Nagelkerke):Proportion of variance explained in the dependent variables.

**Table 4b:** Linear regression analysis results: associations of anti-TPO and TSH with nodule number, ferritin, and NLR.

Dependent Variable	Independent Variable	B	S.E.	β (Beta)	*p*	Confidence Interval 95 C.I.for EXP(B)			Nagelkerke R^2^
						Lower		Upper	
Nodule number	Anti-TPO (per 10 IU/mL)	-0.01	0.01	-0.161	**0.045**	-0.03	0.01		0.026
Ferritin (µg/L)	Anti-TPO (per 10 IU/mL)	0.69	0.32	0.178	**0.034**	0.05	1.32		0.032
NLR	TSH (per 10 mIU/L)	0.47	0.24	0.161	**0.05**	0.01	0.94		0.026

**Note:** *P* values that are statistically significant have been highlighted in bold for emphasis. **Abbreviations:** B: Unstandardized path coefficient, β (Beta): Standardized path coefficient, R^2^: Proportion of variance explained in the dependent variables.

## Data Availability

The analyzed data sets generated during the study are available from the corresponding author upon reasonable request.

## LIST OF ABBREVIATIONS

Anti-TPO: Anti-Thyroid Peroxidase
LT4: Levothyroxine
TSH: Thyroid-Stimulating Hormone
T4: Thyroxine
T3: Triiodothyronine
NLR: Neutrophil-to-Lymphocyte Ratio
SII: Systemic Immune-Inflammatory Index
PIV: Pan-Immune Inflammation Value
CRP: C-Reactive Protein
IL-6: Interleukin-6
TNF-α: Tumor Necrosis Factor-Alpha
ATA: American Thyroid Association
TI-RADS: Thyroid Imaging Reporting and Data Systems

## STANDARDS OF REPORTING

STROBE guidelines were followed.
